# Extremely small wasps independently lost the nuclei in the brain neurons of at least two lineages

**DOI:** 10.1038/s41598-023-31529-4

**Published:** 2023-03-15

**Authors:** Alexey A. Polilov, Kamila D. Hakimi, Anastasia A. Makarova

**Affiliations:** Department of Entomology, Faculty of Biology, Lomonosov Moscow State University, Moscow, 119234 Russia

**Keywords:** Entomology, Zoology

## Abstract

Anucleate animal cells are a peculiar evolutionary phenomenon and a useful model for studying cellular mechanisms. Anucleate neurons were recently found in one genus of miniature parasitic wasps of the family Trichogrammatidae, but it remained unclear how widespread this phenomenon is among other insects or even among different tissues of the same insect species. We studied the anatomy of miniature representatives of another parasitic wasp family (Hymenoptera: Mymaridae) using array tomography and found two more species with nearly anucleate brains at the adult stage. Thus, the lysis of the cell bodies and nuclei of neurons appears to be a more widespread means of saving space during extreme miniaturization, which independently evolved at least twice during miniaturization in different groups of insects. These results are important for understanding the evolution of the brain during miniaturization and open new areas of studying the functioning of anucleate neurons.

## Introduction

The unique phenomenon of the lysis of the cell bodies and nuclei of the neurons in the brain was first described in one of the smallest insects, the parasitic wasp *Megaphragma polilovi*^1^. This phenomenon was subsequently found also in other species of the same genus^2,3^ and the process of denucleation was studied^4^. Anucleate cells have been described in various tissues of animals^5^, including insects^6^. In the miniature dwarf males of the cycliophoran *Symbion pandora*, lysis of the nuclei has been described in various tissues^7^. The survival of axons separated from the cell bodies of neurons has been described for cell cultures^8^. However, anucleate neurons are known to date only in representatives of the genus *Megaphragma* (Hymenoptera: Trichogrammatidae), but it remained unclear how common this phenomenon is among other insects. The aim of this study was to examine the structure of the brain in some of the smallest insects of the family Mymaridae (Hymenoptera) and determine whether they possess anucleate neurons.

## Results and discussion

**Figure 1:**
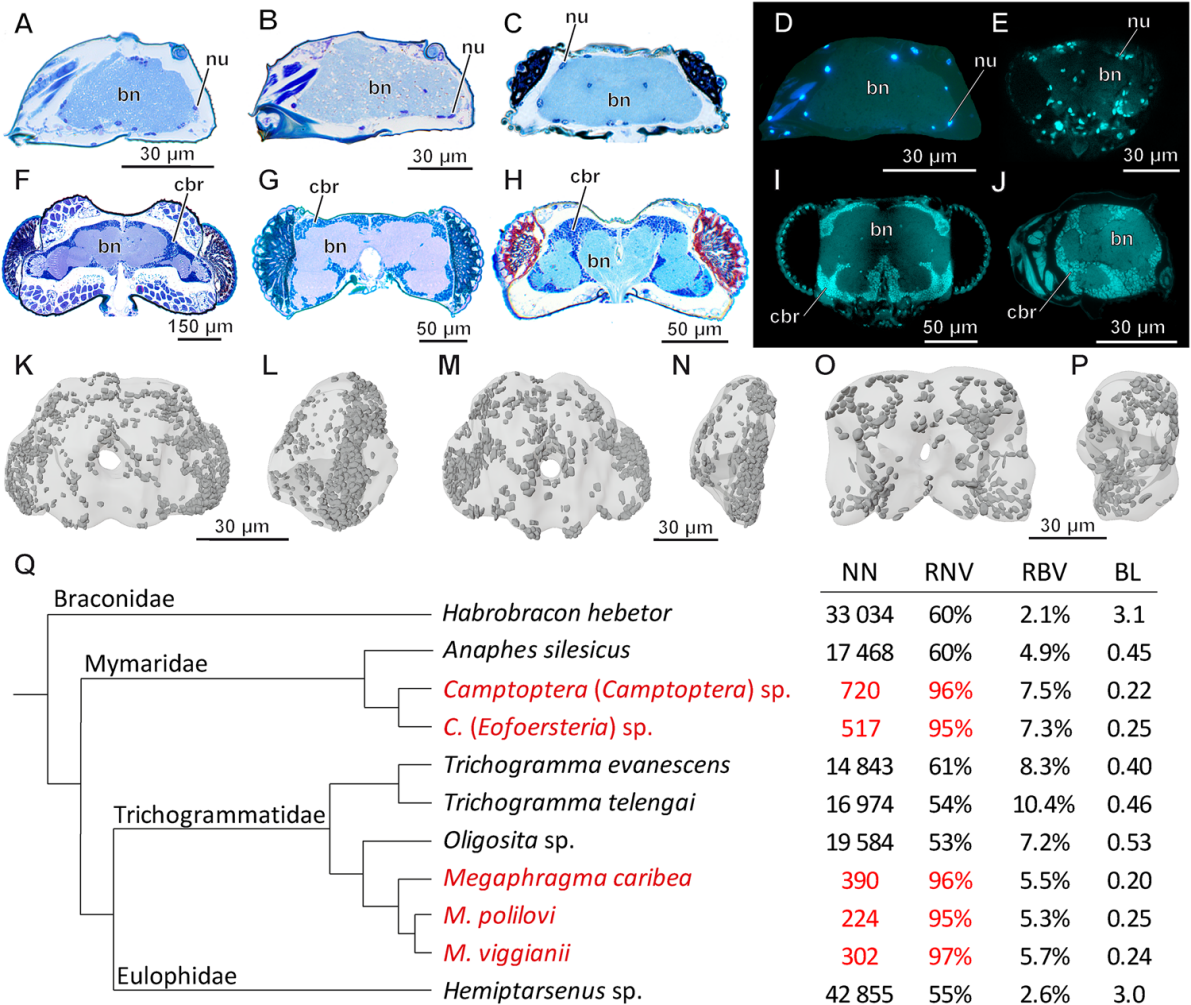
Structure of the brain and its main morphometric characteristics in various Hymenoptera. (**A**–**J**) Sections of the head in species with anucleate neurons (**A**–**E**) and with well-developed cell body rinds (**F**–**J**); (**A**–**C**, **F**–**H**) bright-field microscopy, toluidine blue and pyronine; (**D**,**E**,**I**,**J**) fluorescence microscopy, Dapi; (**A**,**B**,**D**,**J**) sagittal sections; (**C**,**F**–**G**) frontal sections; (**E**,**I**) transverse sections; (**K**–**P**) 3D reconstructions of the brain (nuclei gray and non-transparent, neuropil semitransparent); (**K**,**M**,**O**) frontal view; (**L**,**N**,**P**) lateral view; (**A**,**D**,**K**,**L**) *Camptoptera* (*Camptoptera*) sp.; (**B**,**M**,**N**) *C*. (*Eofoersteria*) sp.; (**C**) *Megaphragma polilovi*; (**E**,**O**,**P**) *M*. *viggianii*; (**F**) *Hemiptarsenus* sp.; (**G**) *Anaphes silesicus*; (**H**) *Trichogramma evanescens*; (**I**), *T*. *telengai*; (**J**) *Oligosita* sp.; (**Q**) phylogeny of the studied insects and their characteristic numbers of nuclei in the brain (NN), percentage of brain volume occupied by the neuropil (RNV), percentage of the body volume occupied by the brain (RBV), and body length in mm (BL): the values are partially taken from previously published studies^1,3,4,10,23,24^; *bn* brain neuropil, *cbr* cell body rind, *nu* nuclei of brain cells.

Study of the structure of the brain in two species of *Camptoptera* (Hymenoptera: Mymaridae) using array tomography shows that their brains do not have a the cell body rind (layer consisting of cell bodies and nuclei) as in other insects (Fig. 1A–P) and contain about 600 nuclei, which is much fewer than in other insects and close to the number of nuclei characteristic of species of the genus *Megaphragma*^4^. At the same time, the relative volume of the neuropil in the studied species of *Camptoptera* is about 95%, which also fundamentally distinguishes them from other insects, which are characterized by a constant ratio of the volume of the neuropil and the cell body rind, 3:2^9^ and similar to that found in *Megaphragma* (Fig. 1Q). These results show that the phenomenon of the lysis of the cell bodies and nuclei of neurons, previously described in *Megaphragma* (Trichogrammatidae), also takes place in *Camptoptera* (Mymaridae).

The structure of the central nervous system of insects in general is strongly conserved, and its morphology and ultrastructure barely change during dramatic changes in body size^10^. Since the efficiency of the nervous system is determined by the number of neurons and contacts between them, the relative volume of the central nervous system, especially the brain, increases considerably with decreasing body size so as to maintain the number of cells and the structure that are required for proper functioning (a phenomenon found in many animals, known as Haller's rule^11^. The size of the nervous system, limited by the minimum number of neurons and by the conserved organization, is one of the factors limiting the minimum body size of insects^12^. Some miniature insects solve the problem of an excessively large brain by shifting it from the head into the thorax, and sometimes even into the abdominal segments^10^. Miniature hymenopterans have a highly mobile narrow neck and had to take a completely different evolutionary path. They had to sacrifice the bodies and nuclei of neurons after the formation of the brain at the pupal stage; as a result, the adult insects have nearly anucleate brains, with 95–97% of the cells are represented only by dendrites and axons. Due to the lysis of the cell bodies and nuclei of neurons, the volume of the adult brain is strongly reduced, and the studied representatives of the genera *Megaphragma* and *Camptoptera* provide exceptions to Haller's rule, since the relative volumes of their brains are comparable to or even smaller than those of larger representatives of related groups of Hymenoptera.

Interestingly, no lysis of nuclei or cell bodies can be found in the peripheral nervous system of miniature hymenopterans. Detailed structure of the sense organs of *Camptoptera* remains unknown, but preliminary results show that the receptors of its eyes and antennae have nuclei. It has been shown for other miniature hymenopterans that all their receptor cells have nuclei, even in those species that have anucleate neurons^13–16^.

Our finding shows that a similar variant of saving space due to the lysis of the cell bodies and nuclei of neurons in the brain and other parts of the central nervous system evolved at least twice in the course of insect evolution. At the same time, miniature insects retain complex forms of behavior and locomotion, which shows that the anucleate neurons remain functional. This raises the important question of how common this phenomenon is in other miniature insects and other miniature arthropods, e.g., hymenopterans of other families (Mymarommatidae, Scelionidae, etc.), mites (Eriophyidae, Microdispidae, etc.), and crustaceans (Chydoridae, Basipodellidae, etc.). A number of other important issues also remain unresolved. First, the mechanisms and control of the process of lysis of the cell bodies and nuclei of neurons remain unknown. And, second, the most important question is how efficiently the dendrites, axons, and remaining nuclei of the neurons function, and what cellular molecular mechanisms provide for their functioning.

Miniature insects can become important model species for neuroscience^17^. Thus, further comprehensive studies of the structure and functioning of the brain in various miniature arthropods is of great fundamental importance for understanding the general principles of the working and evolution of the animal brain.

## Materials and methods

Adults of *Camptoptera* (*Camptoptera*) sp., *C*. (*Eofoersteria*) sp. (Hymenoptera: Mymaridae), and *Oligosita* sp. (Trichogrammatidae) were studied using array tomography. The material was fixed in FAE (formaldehyde, acetic acid, ethanol) and preserved in 70% ethanol, then dehydrated by passing through a series of alcohols of increasing concentration and acetone and embedded in Araldite M. A series of longitudinal sections 0.5 µm thick were made from the obtained blocks on a Leica RM2255 microtome and mounted on glass slides. The resulting preparations were covered with Slowfade medium with Dapi and photographed on an Olympus BX43 fluorescent microscope with a Tucsen FL20 camera in the ultraviolet channel. After that, Slowfade was washed with distilled water and the preparations were stained with toluidine blue and pyronine and then photographed under the same microscope in transmitted light. Section photographs were aligned using the FEI Amira 2019 software (Thermo Fisher Scientific Inc., USA). Aligned and calibrated image stacks were loaded into Bitplane Imaris 9.5 (Oxford Instruments, Switzerland) for further reconstruction. All structures were segmented manually using the “Surfaces” function. The resulting reconstructions were processed in Blender 2.9 (Blender Foundation, Netherlands) using the functions of surface smoothing and rendering (Fig. 1K–P). The volumes of the structures were calculated using 3D reconstructions in the Bitplane Imaris statistical module. The methods were described in more detail earlier^3^.

In the absence of a complete species-level phylogeny of Hymenoptera, we used a combination of the phylogenies of Parasitoida^18^, Chalcidoidea^19,20^, and Trichogrammatidae^21,22^.

## Data Availability

All data are included in the manuscript. Original array tomography images available from the corresponding author on reasonable request.
